# Decomposition of Reinforcement Learning Deficits in Disordered Gambling via Drift Diffusion Modeling and Functional Magnetic Resonance Imaging

**DOI:** 10.5334/cpsy.104

**Published:** 2024-03-20

**Authors:** Antonius Wiehler, Jan Peters

**Affiliations:** 1 Department of Systems Neuroscience, University Medical Centre Hamburg-Eppendorf, Hamburg, Germany; 2 Institut du Cerveau et de la Moelle épinière (ICM), INSERM U 1127, CNRS UMR 7225, Sorbonne Universités Paris, France; 3 Department of Psychology, Biological Psychology, University of Cologne, Cologne, Germany

**Keywords:** Gambling disorder, problem gambling, reinforcement learning, drift diffusion model, feedback-based learning

## Abstract

Gambling disorder is associated with deficits in reward-based learning, but the underlying computational mechanisms are still poorly understood. Here, we examined this issue using a stationary reinforcement learning task in combination with computational modeling and functional resonance imaging (fMRI) in individuals that regular participate in gambling (n = 23, seven fulfilled one to three DSM 5 criteria for gambling disorder, sixteen fulfilled four or more) and matched controls (n = 23). As predicted, the gambling group exhibited substantially reduced accuracy, whereas overall response times (RTs) were not reliably different between groups. We then used comprehensive modeling using reinforcement learning drift diffusion models (RLDDMs) in combination with hierarchical Bayesian parameter estimation to shed light on the computational underpinnings of this performance deficit. In both groups, an RLDDM in which both non-decision time and decision threshold (boundary separation) changed over the course of the experiment accounted for the data best. The model showed good parameter and model recovery, and posterior predictive checks revealed that, in both groups, the model accurately reproduced the evolution of accuracies and RTs over time. Modeling revealed that, compared to controls, the learning impairment in the gambling group was linked to a more rapid reduction in decision thresholds over time, and a reduced impact of value-differences on the drift rate. The gambling group also showed shorter non-decision times. FMRI analyses replicated effects of prediction error coding in the ventral striatum and value coding in the ventro-medial prefrontal cortex, but there was no credible evidence for group differences in these effects. Taken together, our findings show that reinforcement learning impairments in disordered gambling are linked to both maladaptive decision threshold adjustments and a reduced consideration of option values in the choice process.

## Introduction

Gambling disorder is a behavioral addiction that shares core neurobiological features with substance-use-disorders (Fauth-Bühler et al., 2017). Neuroimaging studies have often reported functional and to a lesser degree structural alteration in regions of the reward system in disordered gambling (Clark et al., 2019), in particular in the ventral striatum and the ventromedial prefrontal cortex (vmPFC), regions implicated in reinforcement learning and reward valuation (Bartra et al., 2013; Clithero & Rangel, 2014). However, as summarized extensively in a recent review (Clark et al., 2019), the directionality of dysregulation in these circuits in gambling disorder is mixed, with studies reporting both increases and decreases in responses. These inconsistencies likely depend on task-specific and contextual effects (Leyton & Vezina, 2013; Miedl et al., 2012, 2014), as extensively discussed previously (Balodis et al., 2012; Leyton & Vezina, 2012; van Holst, Veltman, van den Brink, et al., 2012).

Behaviorally, gambling disorder is characterized by maladaptive decision-making in a range of laboratory tasks. This includes increased temporal discounting (i.e. an increased preference for smaller-sooner over larger-later rewards) (Alessi & Petry, 2003; Dixon et al., 2003; Holt et al., 2003; MacKillop et al., 2011; Miedl et al., 2012; Wiehler & Peters, 2015), and increased risk-taking (Ligneul et al., 2012; Miedl et al., 2012; Wiehler & Peters, 2015). There is also evidence of impairments in feedback-based learning tasks in disordered gambling, such as on the Wisconsin Card Sorting Test (WCST) (Alvarez-Moya et al., 2010; Boog et al., 2014; Goudriaan et al., 2005; Hur et al., 2012; Ledgerwood et al., 2012; Zhou et al., 2016). Likewise, gambling disorder is associated with impairments in probabilistic reversal learning (Boog et al., 2014; de Ruiter et al., 2009), and reductions in directed (strategic) exploration in reinforcement learning (RL) (Wiehler et al., 2021). Although there is some heterogeneity across studies with respect to reversal learning impairments, the general directionality of these effects is quite consistent in the literature (van Timmeren et al., 2018).

From a computational reinforcement learning perspective (Sutton & Barto, 2018), such reversal learning impairments in gambling disorder could arise due to changes in several different processes. On the one hand, impairments could be due to response perseveration, where previous actions are repeated irrespective of learned values. Additionally, however, reinforcement learning requires balancing exploration (choosing options with unknown value for information gain) and exploitation (choosing options with known value for reward maximization) (Schulz & Gershman, 2019; Sutton & Barto, 2018; Wilson et al., 2021). Gambling disorder is linked to a reduction in directed exploration (Wiehler et al., 2021), and learning impairments could therefore also be due to reduced exploration. Finally, lower learning rates, an overall a reduced consideration of option values in the decision process, or more liberal decision thresholds (e.g. a focus on speed rather than accuracy) could likewise underlie impairments in disordered gambling (Hales et al., 2023).

Traditionally, RL models account for trial-wise categorical decisions by assuming that choices stochastically depend on the values of the available options. These values are learned via e.g. the delta learning rule, where values are updated based on reward prediction errors (Sutton & Barto, 2018). This learning rule is then combined with a choice rule such as softmax action selection (Sutton & Barto, 2018), where the slope parameter indexes the “value-dependency” or “stochasticity” of decisions with respect to the values implied by a given model. However, such choice rules are agnostic with respect to the computational processes underlying changes in “stochasticity” (Pedersen et al., 2017).

For this reason, recent work has begun to take the distribution of choice response times (RTs) into account. Sequential sampling models such as the drift diffusion model (DDM) (Forstmann et al., 2016; Ratcliff & McKoon, 2008) are widely used in perceptual decision-making. These models assume that choices arise from a noisy evidence accumulation process that terminates as soon the accumulated evidence exceeds a threshold. In its simplest form, the DDM has three free parameters. The drift rate *v* reflects the average rate of evidence accumulation. The decision threshold (boundary separation) parameter *α* governs the response threshold, and thus controls the speed-accuracy trade-off – a lower threshold emphasizes speed over accuracy, whereas the reverse is true for a higher threshold. The non-decision time *τ* models perceptual and/or motor components or the RT that are unrelated to the evidence accumulation process. If one assumes that the quality of evidence in favor of a decision option is reflected in the trial-wise drift rate (via some *linking function* (Miletić et al., 2020)), the DDM can be used to model trial-wise decisions in reinforcement learning (Fontanesi, Gluth, et al., 2019; Pedersen et al., 2017; Shahar et al., 2019) and value-based decision-making more generally (Peters et al., 2020; Peters & D’Esposito, 2020; Wagner et al., 2020). The benefits of such an approach over softmax choice rules are both of technical and theoretical nature. In technical terms, inclusion of RTs during model estimation improves parameter recovery and reliability (Ballard & McClure, 2019; Shahar et al., 2019), which is of particular relevance when the number of observations is small, e.g. when working with clinical samples. In theoretical terms, a combined reinforcement learning DDM (RLDDM) is not only a more complete model, as it accounts for both binary decisions and RTs, but also allows for a more fine-grained analysis of the dynamics underlying the decision process. For example, choices could be more random or stochastic due to a reduced impact of values on drift rates or due to a more liberal decision threshold (lower boundary separation). RLDDMs can dissociate these different possibilities, whereas softmax choice rules only contain a single “stochasticity” parameter, and thus cannot disentangle effects of value-dependency from threshold changes. DDMs can reveal alterations in decision-making following prefrontal cortex damage (Peters & D’Esposito, 2020) and following pharmacological dopamine challenges (Chakroun et al., 2023; Peters et al., 2020; Wagner et al., 2020). Specifically, increasing dopamine neurotransmission via a pharmacological challenge (Chakroun et al., 2023) or via supplementation with the catecholamine precursor tyrosine (Mathar et al., 2022) resulted in reduced decision thresholds. RLDDMs can also reveal pharmacological effects on learning in attention-deficit hyperactivity disorder (Pedersen et al., 2017) and contextual effects in reinforcement learning (Fontanesi, Gluth, et al., 2019; Fontanesi, Palminteri, et al., 2019). More generally, RLDDMs, and computational approaches in general, might provide novel insights into mechanisms underlying disordered gambling (Hales et al., 2023).

Here we leveraged this modeling scheme to comprehensively analyze the computational basis of reinforcement learning deficits in gambling disorder. A gambling group (n = 23) and a matched control group (n = 23) performed a stationary RL task (Chakroun et al., 2023; Pessiglione et al., 2006) while brain activity was measured using functional magnetic resonance imaging (fMRI). The same participants also completed a restless four-armed bandit task, the results from which have been published earlier (Wiehler et al., 2021). In the stationary RL task, model-agnostic analyses revealed a substantial reduction in accuracy in the gambling group. Hierarchical Bayesian computational modeling revealed that an extension of previously proposed RLDDMs (Fontanesi, Gluth, et al., 2019; Miletić et al., 2020; Pedersen et al., 2017) that allowed both non-decision times and decision thresholds to vary over the course of learning accounted for the data best. This model showed good parameter and model recovery, and in both groups accounted for the evolution of accuracies and RTs over the course of learning. In this RLDDM, reduced performance in the gambling group was linked to a more rapid reduction in decision thresholds over time, and a reduced impact of value differences on the drift rate. FMRI replicated core effects of prediction error and value signaling in ventral striatum and ventro-medial prefrontal cortex across groups, but did not reveal credible evidence for group differences in these effects.

## Methods

### Participants

In total, n = 23 individuals that regularly participate in gambling, and n = 23 matched controls took part in the study. All participants provided informed written consent prior to participation, and the study procedure was approved by the local institutional review board (Hamburg Board of Physicians, project code PV4720). Participants were recruited via postings in local internet bulletin boards, and reported no history of neurological or psychiatric disorder except for depression. No participants were currently undergoing any psychiatric treatment. Current drug abstinence on the day of testing was verified via a urine drug test.

The demographic and clinical characteristics of the groups have been reported in detail elsewhere (Wiehler et al., 2021). In short, groups were matched on age (M[SD] gambling: 25.91 [6.47], controls: 26.52 [5.92], t = –.33, p = .74), gender (all participants were male), self-reported smoking according to the Fagerström Test for Nicotine Dependence (FTND) (Heatherton et al., 1991) (M[SD] gambling: 2.14 [2.58], controls: 1.83 [2.18], t = .44, p = .66), self-reported alcohol use according to the Alcohol Use Disorders Test (AUDIT) (Saunders et al., 1993) (M[SD] gambling: 6.09 [7.14], controls: 6.52 [4.57], t = –.24, p = .81) and education (school years M[SD] gambling: 11.64 [1.77], controls: 11.91 [1.35], t = –.60, p = .55). The gambling group exhibited higher depressive symptoms according to self-report (Beck Depression Inventory (BDI-II) (Beck et al., 1996), M[SD] gambling: 15.41 [11.41], controls: 7.61 [7.94], t = 2.69, p = .01).

DSM-5 criteria were assessed via a semi-structured interview by a researcher with basic clinical training (A. W.). Sixteen individuals from the gambling group fulfilled four or more DSM-5 criteria for gambling disorder (i.e. meeting the DSM-5 threshold for disordered gambling). Seven individuals fulfilled one to three DSM-5 criteria. The severity of problem gambling symptoms was further characterized using two self-report scales, the German “Kurzfragebogen zum Glücksspielverhalten” (KFG) (Petry, 1996), where the gambling group exhibited substantially higher scores (M[SD] gambling: 25.90 [14.15], controls: 0.58 [0.32], t = 8.55, p < .001) and the South Oaks Gambling Screen (SOGS) (Lesieur & Blume, 1987), where the gambling group likewise showed substantially higher scores (M[SD] gambling: 8.64 [4.46], controls: 0.21 [0.54], t = 8.99, p < .001).

Due to an incidental finding during fMRI, which may have impacted spatial normalization, one control participant was excluded from the imaging data analysis. This participant was however retained for all behavioral and modeling analyses.

### Reinforcement learning task

**Figure 1:**
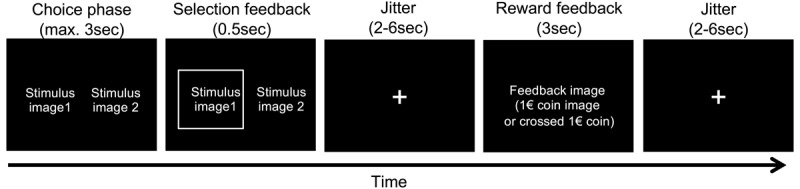
Illustration of a single trial from the reinforcement learning task. Stimuli were presented for a maximum of 3sec, during which participants were free to make their selection. The selection was then highlighted for 500 ms, followed by a jitter of variable duration (2–6sec). Reward feedback was then presented for 3sec, followed by another jitter of variable duration (2-6sec). Stimuli consisted of two pairs of abstract fractal images (80% vs. 20% reinforcement rate), which were presented in randomized order, and participants completed 30 trials per pair.

Following completion of our previously reported restless four-armed bandit task (Wiehler et al., 2021), participants had a short break inside the scanner. Then they performed 60 trials in total of a stationary reinforcement learning task (Chakroun et al., 2023; Pessiglione et al., 2006) using two pairs of stimuli (n = 30 trials per pair). Per pair, one stimulus was associated with a reinforcement rate of 80% (optimal stimulus) whereas the other was associated with a reinforcement rate of 20% (suboptimal stimulus). Options were randomly assigned to the left/right side of the screen, and trials from the two option pairs were presented in randomized order. Participants had three seconds to choose one of the two stimuli via button press (see Figure 1). Participants received binary feedback, either in the form the display of a 1€ coin (*reward* feedback, see Figure 1) or as a crossed 1€ coin (*no reward* feedback). A jitter of variable duration (2–6sec, uniformly distributed) was included following presentation of the selection feedback and following presentation of the reward feedback (see Figure 1). Prior to scanning, participants performed a short practice version of the task in order to familiarize themselves with the task and the response deadline. Participants received 10% of the collected 1€ coins as an additional performance-contingent financial compensation.

### Model-agnostic statistical analyses

Model-agnostic measures (accuracy, median RT) were analyzed using Bayesian Wilcoxon Rank-Sum tests as impletemented in JASP (Wagenmakers et al., 2018) (Version 0.l6.3).

### Q-learning model

We applied a simple Q-learning model (Sutton & Barto, 2018) to formally model the learning process. Here, participants are assumed to update the value (Q-value, Eq. 1) of the chosen action *i* based on the reward prediction error *δ_t_* computed on each trial *t* as the difference between the obtained reward *r_t_* and the expected reward *Q_i,t_*, weighted by the learning rate *η*:


1 \[
{Q_{i,t + 1}} = {Q_{i,t}} + {\eta}^{*}{\delta _t}
\]

2 \[
{\delta _t} = {r_t} - {Q_{i,t}}
\]

Q-values of unchosen actions remain unchanged. Q-values were initialized with values of 0.5. As learning from positive and negative feedback is thought to depend on distinct striatal circuits (Frank et al., 2004; Maia & Frank, 2011), we also examined models with separate learning rates (*η*_+_, *η*_–_) for positive vs. negative prediction errors. Learning rates were estimated in standard normal space [–3, 3] and back-transformed to the interval [0, 1] via the inverse cumulative normal distribution function.

### Softmax action selection

Softmax action selection models the choice probability of the chosen action *i* on trial *t* as a sigmoid function of the Q-value difference (Sutton & Barto, 2018) between the optimal and suboptimal options:


3 \[
P{\left(i \right)_t} = \frac{1}{{1 + {\mathrm{exp}} (-[{Q_{optimal}} - {Q_{suboptimal}}]*\beta)}}
\]

The inverse temperature parameter *β* models the degree to which choice probabilities depend on Q-values, such that choices are random for *β* = 0, and increasingly depend on the Q-value differences between options as *β* increases.

### Reinforcement learning drift diffusion models (RLDDMs)

We next set out to more comprehensively analyze choice dynamics underlying learning performance. To this end, we examined a set a reinforcement learning drift diffusion models (Chakroun et al., 2023; Fontanesi, Gluth, et al., 2019; Pedersen et al., 2017) (RLDDMs) in which the DDM replaces softmax action selection as the choice rule (Miletić et al., 2020). These models can account for the full response time (RT) distributions associated with decisions, and thus provide additional information regarding the dynamics of the choice process.

The upper response boundary was defined as selection of the optimal (80% reinforced) stimulus, whereas the lower response boundary was defined as selection of the suboptimal (20% reinforced) stimulus. RTs for choices of the suboptimal option where multiplied by –1 prior to model estimation, and we discarded for each participant the fastest 5% of trials. The reason is that fast responses that fall beyond the leading edge of the RT distribution can force the estimated non-decision time to adjust to accommodate these values, which can negatively impact model fit. In a null model without a learning component (DDM_0_), the RT on each trial *t* is then distributed according to the Wiener First Passage Time (*wfpt*):


4 \[
R{T_t}\sim wfpt(\alpha,\tau,\;z,v)
\]

Here the decision threshold parameter *α* regulates the speed-accuracy trade-off, such that smaller values of *α* lead to faster but less accurate responses. The drift rate *v* reflects the quality of the evidence, such that greater values of *v* give rise to more accurate and faster responses. Note that in this model *v* is constant and not affected by learning. The non-decision time *τ* models RT components related to motor and/or perceptual processing and unrelated to the evidence accumulation process. The starting point parameter *z* models a bias towards one of the response boundaries. We fixed *z* at .5 as options were presented in randomized order on the left vs. right side of the screen, and an *a priori* bias towas optimal or suboptimal choices is not plausbile in this learning setting.

Following earlier work (Chakroun et al., 2023; Pedersen et al., 2017) we then incorporated the learning process (Equations 1 and 2) in the DDM by setting trial-wise drift rates to be proportional to the difference in Q-values between optimal and suboptimal options using a simple linear linkage function (Chakroun et al., 2023; Miletić et al., 2020; Pedersen et al., 2017):


5 \[
{v_t} = {v_{coeff}}*({Q_{optimal}} - {Q_{suboptimal}})
\]

*v_coeff_* models the degree to which trial-wise drift rates scale with the value difference between options. The intuition is that as Q-value differences increase, accuracy should increase, and RTs should decrease. Conversely, when Q-values are similar (and response conflict is high) choices should be both more random and slower. Note that we also examined a non-linear mapping scheme proposed in earlier work (Fontanesi, Gluth, et al., 2019), but, as in earlier related work (Chakroun et al., 2023) these models failed to converge in our data. This is likely attributable to the lower trial numbers in the present study compared to previous implementations of non-linear drift rate scaling (Fontanesi, Gluth, et al., 2019; Peters & D’Esposito, 2020; Wagner et al., 2020).

We also examined two further extensions of the RLDDM that might capture additional RT effects unrelated to the learning process. These extensions were motivated by the observation that in the gambling group, RTs decreased over the course of the experiment, but this effect was only in part attributable to learning, such that models with constant *α* and *τ* did not fully reproduce RT changes in the gambling group (see posterior predictive checks below). Therefore, we examined whether allowing decision threshold (Fontanesi, Gluth, et al., 2019; Pedersen et al., 2017) and/or non-decision-time to vary over the course of the experiment according to a power function (as in previous work (Fontanesi, Gluth, et al., 2019; Pedersen et al., 2017)) could account for these effects. For the case of decision threshold *α* that varies across trials *t*, this yields


6 \[
{\alpha _t} = {\alpha _0}*{t^{{\alpha _{exp}}}}
\]

In the same vein, for the case of non-decision-time *τ* that varies across trials *t*, this yields


7 \[
{\tau_t} = {\tau_0}*{t^{{\tau_{exp}}}}
\]

Decision threshold and non-decision time start at values of *α*_0_ and *τ*_0_ on trial 1. Parameter values then change over trials according power functions with exponents *α_exp_* and *τ_exp_*. The first case (Eq. 6) captures the idea that, over time, participant’s decison thresholds might decrease due to e.g. impatience, fatigue or boredom with the task. The second case (Eq. 7) corresponds to the idea that motor and/or perceptual processes might speed up over time, e.g. due to practice effects, increased familiarity with the task or impatience.

Therefore, the model space included the null model (DDM_0_) and eight variants of the RLDDM, which differed according to learning rates (single vs. dual), decision thresholds (fixed vs. power function) and non-decision times (fixed vs. power function).

### Hierarchical Bayesian models

Models were fit to all trials from all participants, separate for each group, using a hierarchical Bayesian modeling approach with group-level Gaussian distributions for all parameters. Posterior distributions were estimated using Markov Chain Monte Carlo as implemented in the JAGS software package (Plummer, 2003) (Version 4.3) using the Wiener module for JAGS (Wabersich & Vandekerckhove, 2014) distribution, in combination with Matlab (The MathWorks) and the *matjags* interface (https://github.com/msteyvers/matjags). For group-level means and standard deviations, we defined uniform priors over numerically plausible parameter ranges (see Table 1), and applied identical prior distributions for each group.

**Table 1:** Overview of priors for group means.


PARAMETER	GROUP-LEVEL PRIOR (μ)	GROUP-LEVEL PRIOR (σ)

***α***_0_	*Uniform (.01, 5)*	*Uniform (.0001, 2)*

***α***_exp_	*Uniform (–3, 3)*	*Uniform (.0001, 2)*

***τ***_0_	*Uniform (0.1, 2)*	*Uniform (.0001, 2)*

***τ***_exp_	*Uniform (–3, 3)*	*Uniform (.0001, 2)*

*v_coeff_*	*Uniform (–100, 100)*	*Uniform (.0001, 10)*

***η***_+_, ***η***___	*Uniform (–3,3)*	*Uniform (.0001, 4)*


For each model and group, we ran two chains with a burn-in period of 50k samples and thinning factor of 2. 10k additional samples were then retained for further analysis. Chain convergence was assessed by examining the Gelman-Rubinstein convergence diagnostic \[
\hat R
\] , and values of \[
1 \le \hat R \le 1.01
\] were considered as acceptable for all group-level and individual-subject parameters. Relative model comparison was performed via the Widely Applicable Information Criterion (WAIC) and the estimated log pointwise predictive density (*elpd*) (Vehtari et al., 2017), an approximation of the leave-one-out cross-validation accuracy of the model.

### Parameter recovery simulations

Parameter recovery simulations were conducted to ensure that known parameters underlying the data-generating process could be recovered using our modeling procedures. For this purpose, we simulated 10k full data sets from the posterior distribution of the best-fitting model. Ten of these simulated data sets were randomly selected, and re-fit with the same modeling procedure. Parameter recovery was then assessed in two ways. For subject-level parameters, we examined the correlation between generating and estimated parameters across all ten simulations. For group-level means and standard deviations, we examined whether the estimated 95% highest posterior density intervals contained the true generating parameter value.

### Model recovery simulations

To ensure that the true data-generating model could be identified using our modeling procedures, model recovery analyses were conducted, focusing on the three best-fitting models (RLDDM 4, RLDDM6 and RLDDM8). Twenty full datasets were simulated from each of the three models’ posterior distributions, and re-fit with all nine models from the model space. The percentage of simulations in which the true data-generating model was recovered was then taken as a measure of model recovery.

### Posterior predictive checks

Posterior predictive checks were performed to ensure that the best-fitting model captured key aspects of the data, again using data sets simulated from the model’s posterior distributions. For each simulated data set, we then computed for each group mean RTs and accuracies for bins of ten trials (averaging across 1k randomly selected simulated data sets), and compared these model-predicted values to the observed data per group. Individual-participant posterior pedictive checks were carried out by overlaying simulated and observed individual-participant RT distributions, and by overlaying simulated and observed RT changes over the course of learning via five trial bins.

### Analyses of posterior distributions

Posterior distributions were analyzed in the following ways. Mean group differences along with 95% highest density intervals and posterior probabilities for group differences > 0 are reported, where probabilities exceeding 95% are taken as evidence for an effect. For completeness, we also report directed Bayes Factors (dBFs) that quantify the relative evidence in favour of a group difference < 0 vs. a group difference > 0.

### FMRI data acquisition

MRI data were collected on a Siemens Trio 3T system using a 32-channel head coil. Participants performed a single run of 60 trials in total (following a short break, after completion of our previously reported task (Wiehler et al., 2021)). Each volume consisted of 40 slices (2 × 2 × 2 mm in-plane resolution and 1-mm gap, repetition time = 2.47s, echo time 26 ms). We tilted volumes by 30° from the anterior and posterior commissures connection line to reduce signal drop out in the ventromedial prefrontal cortex and medial orbitofrontal cortex (Deichmann et al., 2003). Participants viewed the screen via a head-coil mounted mirror, and logged their responses via the index and middle finger of their dominant hand using an MRI compatible button box. High-resolution T1 weighted structural images were obtained following completion of the cognitive tasks.

### FMRI preprocessing

All preprocessing and statistical analyses of the imaging data was performed using SPM12 (Wellcome Department of Cognitive Neurology, London, United Kingdom). As in our previous study in this sample (Wiehler et al., 2021), volumes were first realigned and unwarped to account for head movement and distortion during scanning. Second, slice time correction to the onset of the middle slice was performed to account for the shifted acquisition time of slices within a volume. Third, structural images were co-registered to the functional images. Finally, all images were smoothed (8 mm FWHM) and normalized to MNI-space using the DARTEL tools included in SPM12 and the VBM8 template.

### FMRI statistical analysis

Error trials were defined as trials were no response was made, or trials that were excluded from the computational modeling during RT-based trial filtering (see above, recall that for each participant, the fastest 5% of trials were excluded). Following earlier work (Chakroun et al., 2023), three first-level general linear models (GLMs) were examined. GLM1 used the following regressors:

1. onset of the decision option presentation
2. onset of the decision option presentation modulated by chosen – unchosen Q-value
3. onset of the decision option presentation modulated by (chosen – unchosen Q-value)^2^
4. onset of the feedback presentation
5. onset of the feedback presentation modulated by model-based prediction error
6. onset of the decision option presentation for error trials
7. onset of the feedback presentation for error trials.

In GLM2, chosen – unchosen value was replaced with the average Q-value across options.

GLM3 used the following regressors:

1. onset of the decision option presentation
2. onset of the decision option presentation modulated by chosen – unchosen value
3. onset of the decision option presentation modulated by (chosen – unchosen value)^2^
4. onset of the feedback presentation for positive prediction errors
5. onset of the feedback presentation for negative prediction errors
6. onset of the feedback presentation for error trials.

Following earlier work using this task (Chakroun et al., 2023; Pessiglione et al., 2006), Q-values and prediction errors were computed using the posterior group-mean learning rates from the best-fitting final hierarchical Bayesian model (RLDDM8). Parametric modulators were *z*-scored within-subject prior to entering them into the first level model (Lebreton et al., 2019). Single-subject contrast estimates were then taken to a second-level random effects analysis using the two-sample t-test model as implemented in SPM12. At the second level, the following *z*-scored covariates were included: age, depression as assessed via the Beck Depression Inventory II (BDI) (Beck et al., 1996), smoking behavior as assessed via the Fagerström Test for Nicotine Dependence (FTND) (Heatherton et al., 1991) and alcohol use as assessed via the Alcohol Use Disorders Identification Test (AUDIT) (Saunders et al., 1993).

All contrasts are displayed at p < .001 (*uncorrected*) with k >= 10 voxels, and correction for multiple comparisons using the family-wise error rate (FWE) followed the same approach as in our earlier work (Chakroun et al., 2023) and used a single region-of-interest (ROI) mask provided by the Rangel Lab (https://www.rnl.caltech.edu/resources/index.html) that is based on two meta-analysis of reward valuation effects (Bartra et al., 2013; Clithero & Rangel, 2014). This mask covers core areas involved in reward processing, including bilateral ventral striatum, ventromedial prefrontal cortex, anterior cingulate cortex and posterior cingulate.

## Results

Behavioral data analysis and computational modeling proceeded in the following steps. We first analyzed model-free performance measures. Next, we carried out a detailed model comparison of a set of candidate reinforcement learning drift diffusion models (RLDDMs) and identified the best-fitting model. We then ran parameter and model recovery analyses to ascertain that the true data-generating parameters could be recovered, and ran posterior predictive checks to ensure that key patterns in the data could be reproduced by the best-fitting model. Finally, we examined the parameter posterior distributions and compared them between groups, before moving to the analysis of the fMRI data.

### Model-agnostic analysis

**Figure 2:**
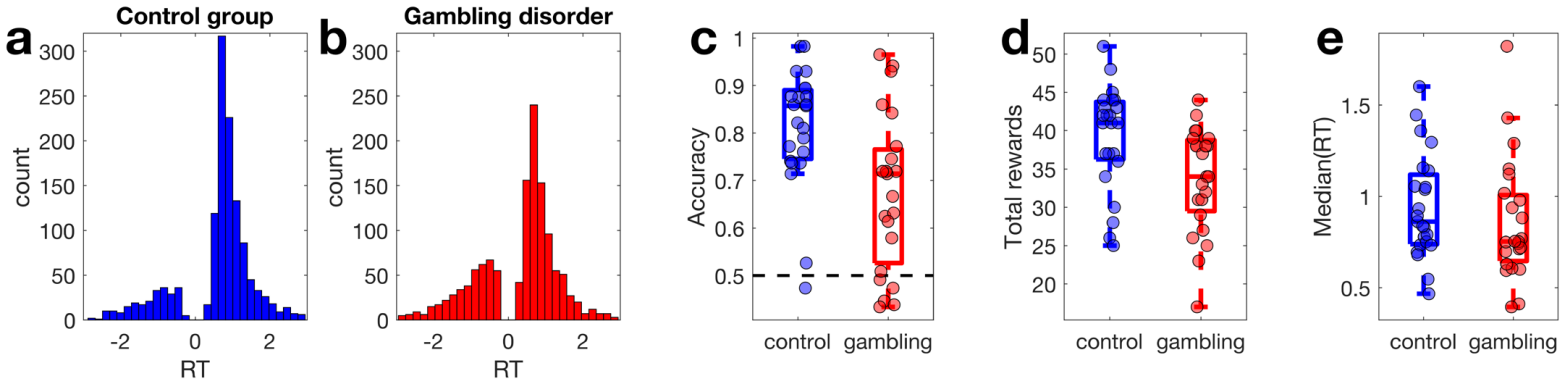
Response time distributions (RT, in seconds) in the control group **(a, blue)** and the gambling disorder group **(b, red)** with choices of the suboptimal options coded as negative RTs. **c**: Accuracy per group (chance level is 0.5). **d**: Total rewards earned per group. **e**: Median RTs per group.

RT distributions per group are shown in Figure 2a and 2b, with choices of the suboptimal option coded as negative RTs. While control group participants selected the optimal stimulus on around 80% of trials (Figure 2c), participants from the gambling group only made around 68% correct choices. A Bayesian Wilcoxon Rank sum test confirmed moderate evidence for group differences in accuracy (BF10 = 6.67, Figure 2c) and total reward obtained (BF10 = 3.94, Figure 2d). For median RTs, in contrast, a Bayesian Wilcoxon Rank Sum test revealed anecdotal evidence for the null model (BF01 = 1.87, Figure 2e).

### Model comparison

We next compared a range of computational models (see methods section). As a reference, we first fit a null model (DDM_0_) without a learning component. Next, a set of reinforcement learning DDMs (RLDDMs) was examined that all included a linear mapping from Q-value differences to trial-wise drift rates (Chakroun et al., 2023; Miletić et al., 2020; Pedersen et al., 2017) (see Eq. 5). This modeling scheme incorporates the intuition that successful learning should decrease RTs and increase accuracies, and that accuracy should be higher and RTs shorter when making easier choice (i.e. when Q-value differences are larger). The model space included models with single vs. dual learning rates η (for positive vs. negative prediction errors), and models with fixed vs. modulated decision threshold *α* and non-decision times *τ* (see Eq. 6 and 7), yielding a total of eight RLDDMs (see Table 2).

**Table 2:** Model comparison results, separately per group. We examined reinforcement learning drift diffusion models (RLDDMs) with single vs. dual learning rates (η) and fixed vs. modulated non-decision times (*τ*) and decision threshold (*α*), as well as a null model without learning (DDM_0_). Model comparison used the estimated log pointwise predictive density (-elpd)(Vehtari et al., 2017). We also report the 95% CI of the difference in -elpd between each model and the best-fitting model (-elpd_diff_).


MODEL	η	τ	*α*	CONTROLS			GAMBLERS		
	
				-*elpd*	-*elpd*_*diff*_	*RANK*	-*elpd*	-*elpd*_*diff*_	*RANK*

**DDM_0_**	–	Fixed	Fixed	800.2	215.0 [171.6, 258.4]	9	1115.9	107.6 [78.9, 136.3]	9

**RLDDM1**	1	Fixed	Fixed	658.1	72.8 [48.6, 97.1]	8	1055.5	47.3 [27.3, 67.2]	8

**RLDDM2**	1	Fixed	Power	634.3	49.0 [29.3, 80.7]	6	1021.4	13.1 [.4, 25.8]	4

**RLDDM3**	1	Power	Fixed	644.0	58.8 [36.8, 97.1]	7	1027.1	18.8 [3.1, 34.4]	6

**RLDDM4**	1	Power	Power	628.3	43.1 [24.1, 68.7	5	1010.3	2.0 [–9.1, 13.2]	2

**RLDDM5**	2	Fixed	Fixed	615.0	29.7 [15.9, 43.5]	4	1049.0	40.7 [23.8 57.6]	7

**RLDDM6**	2	Fixed	Power	591.4	6.1 [–.1, 12.3]	2	1019.3	11.1 [4.5, 17.6]	3

**RLDDM7**	2	Power	Fixed	599.8	14.5 [4.4, 24.6]	3	1022.4	14.1 [3.4, 24.9]	5

**RLDDM8**	2	Power	Power	585.3	0.0	1	1008.3	0.0	1


Model comparison was performed using the estimated log pointwise predictive density (-elpd) (Vehtari et al., 2017) (Table 2). In both groups, RLDDM8 exhibited the lowest -elpd value. However, the 95% confidence intervals of the -elpd difference between the best model and the second-best models (RLDDM6 in the control group and RLDDM4 in the gambling group) overlapped with zero, indicating that the evidence in favour of RLDDM8 was overall not decisive.

Despite this inconclusive model comparison, we focused all remaining analyses on RLDDM8, for the following reasons. First, in the control group, the overlap in -elpd between RLDDM6 and 8 was numerically very small. Second, model recovery was substantially better for RLDDM8 than RLDDM4 and RLDDM6 (see below). Third, RLDDM4 and 6 are nested versions of RLDDM8. In RLDDM4, positive and negative learning rates are identical, η_+_ = η_–_, and in RLDDM6, *τ_exp_* = 0. In such cases an estimation approach (i.e. examining the posterior distributions of the parameters) may be more informative than relying solely on categorical model comparison (Kruschke, 2015). The reason is that a parameter’s posterior distribution provides the best information regarding the value of a parameter, given the priors and the data, and thus allows for a quantification of the degree of evidence that e.g. learning rates differ, or that *τ_exp_* is different from 0.

### Parameter and model recovery simulations

Parameter recovery analyses were carried out across 10 simulated datasets. Results are provided in Supplemental Figure 1 for RLDDM8 and Supplemental Figure 2 for RLDDM4. All correlations between generating and estimated individual-subject parameters were ≥ .59 (see Supplemental Table 1) and group-level parameters recovered well (Supplemental Figures 1 and 2).

Model recovery analyses were restricted to the best fitting model (RLDDM8) and the two runner-up models (RLDDM4 and RLDDM6). Amonst these models, RLDDM8 exhibited the best model recovery performance (Supplemental Figure 3), such that in 77% of simulations from RLDDM8, this model also provided the best fit amongst all models from the model space.

### Posterior predictive checks

**Figure 3:**
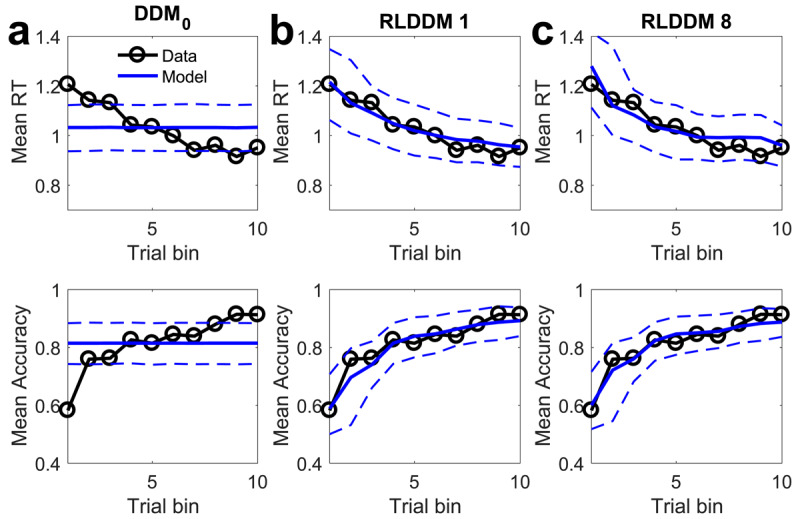
Posterior predictive checks in the control group. Top row: observed RTs over time (black lines) and model predicted RTs (solid blue lines: means, dashed lines: +/– 95% percentiles). Bottom row shows observed accuracies over time (black lines) and model predicted accuracies (solid blue lines: means, dashed lines: +/– 95% percentiles). **a)** DDM_0_ without reinforcement learning. **b)** RLDDM1 with a single learning rate, fixed non-decision time and fixed decision threshold. **c)** RLDDM8 with dual learning rates, modulated non-decision time and modulated decision threshold.

**Figure 4:**
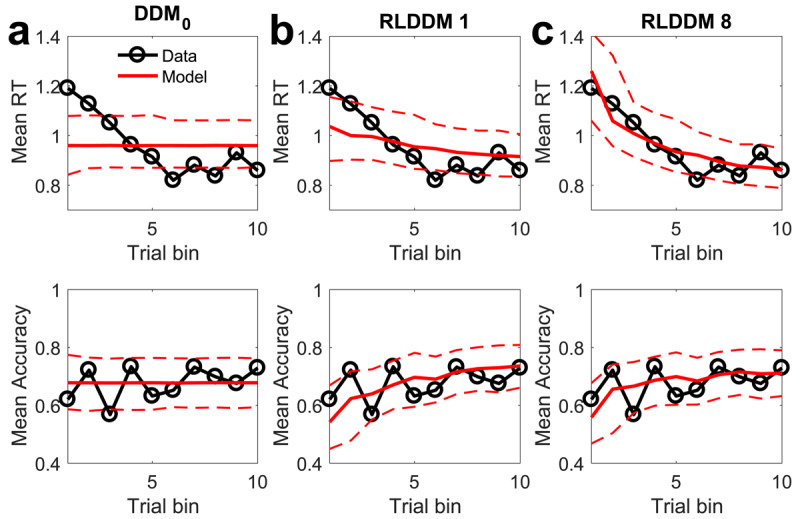
Posterior predictive checks in the gambling disorder group. Top row: observed RTs over time (black lines) and model predicted RTs (solid red lines: means, dashed lines: +/– 95% percentiles). Bottom row shows observed accuracies over time (black lines) and model predicted accuracies (solid red lines: means, dashed lines: +/– 95% percentiles). **a)** DDM_0_ without reinforcement learning. **b)** RLDDM1 with a single learning rate, fixed non-decision time and fixed decision threshold. **c)** RLDDM8 with dual learning rates, modulated non-decision time and modulated decision threshold.

As a model comparison is always relative to a given set of candidate models, we next performed posterior predictive checks to examine the degree to which RLDDM8 accounted for key patterns in the data, in particular with respect to changes in accuracy and RT over the course of learning. For comparison, we included the DDM_0_, and the simplest learning model (RLDDM1), and overlayed mean accuracies and RTs per time bin of simulated and observed data (see methods section), separately for each group. Figure 3 (control group) and Figure 4 (gambling group) depict the observed and model-predicted accuracies and RTs per trial bin.

DDM_0_ predicts constant accuracies and RTs over trials, and as can be seen in Figures 3a and 4a, cannot reproduce the observed learning-related changes. In contrast, RLDDMs predict learning-related increases in accuracy and decreases in RTs over time. Notably, in the control group (Figure 3b, c), both RLDDM1 and RLDDM8 provide a reasonbly good account of both effects on the group level. In contrast, in the gambling disorder group (Figure 4b, c), RLDDM1 provided a poor account of group-level changes in RTs over time, suggesting that RL alone was insufficient to account for the RT reductions over time in the gambling group.

Individual-participant posterior predictive checks confirmed that RLDDM8 provided a good account of individual-participant RT distributions (Supplemental Figures 4 and 5) and RT changes over the course of learning in individual participants (Supplemental Figures 6 and 7).

### Group differences in model parameters

**Figure 5:**
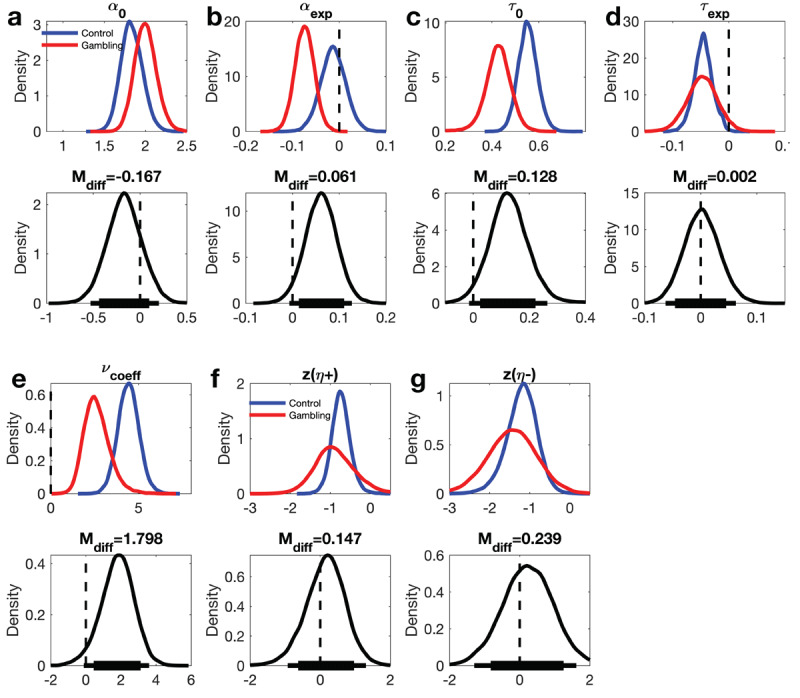
Posterior distributions for RLDDM8 parameters. Upper panels: posterior distributions of parameter group means for the control group (blue) and the gambling group (red). Lower panels: posterior group differences per parameter (control group – gambling disorder group). Solid (thin) horizontal lines in the lower panels denote 85% (95%) highest posterior density intervals.

Next, group differences in RLDDM8 parameters were examined in detail. Posterior distributions of parameter group means as well as group differences are shown in Figure 5 for each RLDDM parameter, and details are provided in Table 3. Three reliable group differences emerged, with posterior probabilities >96% (Table 3): First, α_exp_ was reliably reduced in the gambling group compared to the control group (Figure 5b and Table 3, model-implied decision threshold changes over time for each group and individual are shown in Supplemental Figure 8). The gambling disorder group therefore showed a more rapid reduction in decision thresholds over time than the control group. Second, the offset of the non-decision-time, τ_0_, was reliably lower in the gambling group compared to the control group (Figure 5c and Table 3). Third, the drift rate value modulation, *v_coeff_*, was reliably lower in the gambling group compared to the control group (Figure 5e and Table 3).

**Table 3:** Group differences and within-group effects for all RLDDM8 parameters. M_diff_: mean posterior group difference. P(group diff. > 0): posterior probability that the group difference in a parameter is > 0. dBF (group difference): directional Bayes Factors comparing the evidence for a group difference > 0 to the evidence for a group difference < 0. Within group comparisons: P(effect): posterior probability for an effect (for α_exp_, τ_exp_ and *v_coeff_*, the comparison is *vs*. 0). dBF: directional Bayes Factors comparing the evidence for a parameter value > 0 to the evidence for a parameter value < 0.


	GROUP DIFFERENCES			WITHIN-GROUP COMPARISONS			
	
	*M_diff_*	*P (group diff. > 0)*	*dBF*	*CONTROL GROUP*		*GAMBLING GROUP*	
	
				*P (effect)*	*dBF*	*P (effect)*	*dBF*

α_0_	–.167	18.25%	.29	–			

α_exp_	.061	96.39%	27.60	69.29%	.43	99.98%	.00024

τ_0_	.128	96.91%	27.09	–			

τ_exp_	.002	52.05%	1.12	99.71%	.003	96.10%	.045

*v_coeff_*	1.79	96.40%	25.79	>99.99%	15860	>99.99%	15828

η_+_	.147	62.44%	1.64	–			

η_–_	.239	63.09%	1.66	–			


For comparison, behavioral data were also fitted with a standard softmax choice rule (Eq. 3). Here, the inverse temperature parameter (*β*) was substantially reduced in the gambling group compared to the control group (see Supplemental Figure 10 and Supplemental Table 2). This is consistent with the effects observed for RLDDM8, as both a lower value coefficient of the drift rate and a lower decision threshold would translate to higher levels of decision noise (a lower *β* parameter) in the softmax model.

### FMRI results

**Figure 6:**
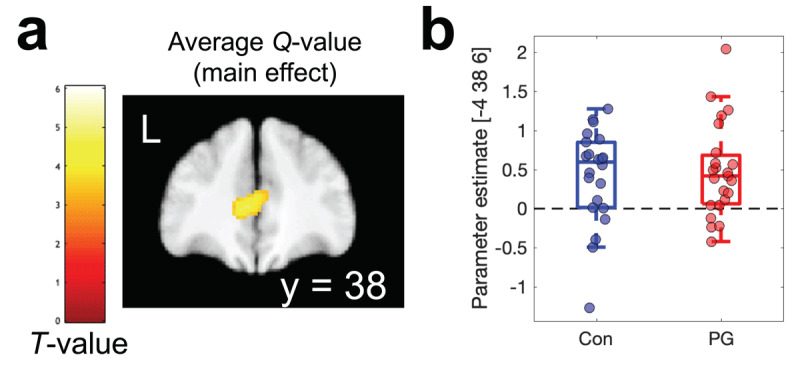
Parametric analyses of model-based average Q-values (GLM2) revealed a robust main effect across groups in the ventro-medial prefrontal cortex **(a)**. Parameter estimates at the peak voxel from **(a)** are shown in **b)**.

In a first step, replication analyses for previously reported effects were conducted, focusing on model-based chosen – unchosen value, and model-based prediction error (based on GLM1 and GLM3) and model-based average Q-value (GLM2). We focused on a single ROI covering areas linked to reward valuation effects based on two meta-analyses (see methods section). This revealed significant main effects across groups for average Q-values in the ventromedial prefrontal cortex (Figure 6 and Table 4). A Bayesian two-sample t-test revealed moderate evidence in favour of the absence of a group effect (BF_01_ = 3.179).

**Table 4:** Replication analyses for model-derived measures (main effects across groups): average Q-value across options, chosen – unchosen Q-value, and model-derived prediction error. Small volume correction for multiple comparisons (SVC) used a single region of interest mask across two meta-analyses (Bartra et al., 2013; Clithero & Rangel, 2014) of reward value effects (see methods section).


CONTRAST/*REGION*	COORDINATES			PEAK T-VALUE	*p*(FWE)_SVC_

Average Q-value					

*vmPFC*	–4	38	6	4.73	.002

Chosen-unchosen value					

*No significant effects in ROI*					

Reward prediction error					

*Left ventral striatum*	–10	6	–10	5.69	<.001

*Right ventral striatum*	12	10	–12	6.77	<.001

*vmPFC*	–4	56	–4	6.26	<.001

*Posterior Cingulate Cortex*	0	–36	–36	4.49	.012


**Figure 7:**
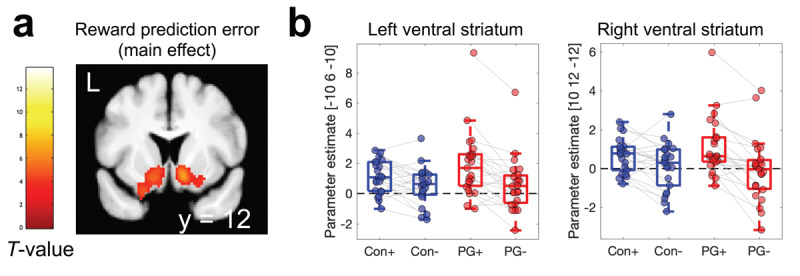
Parametric analysis of model-based reward prediction error (GLM1) revealed a robust main effect across groups in bilateral ventral striatum **(a)**. Parameter estimates at peak voxels in (a) were then extracted from GLM3 to illustrate effects of positive (+) vs. negative (–) prediction errors in each group in both left and right ventral striatum **(b)**.

There were significant effects of model-based prediction error in bilateral ventral striatum, ventro-medial prefrontal cortex and posterior cingulate cortex (Figure 7 and Table 4), whereas no significant effects were observed for chosen – unchosen Q-values in our ROI. Prediction error effects were first identified via parametric modulation in GLM1, and then visualized by extracting separate parameter estimates for positive vs. negative prediction errors from GLM3 (Figure 7b). Statistical analysis of group differences at peak voxels showing main effects of prediction error in GLM1 then used Bayesian repeated measures ANOVAs with the within-subjects factor prediction error sign (positive/negative) and the between-subjects factor group (gambling/control). In both left and right ventral striatum, this revealed decisive evidence for an effect of prediction error sign (Table 5), but only inconclusive evidence for group effects (BF_incl_ < 1) and only anecdotal evidence for the presence of group x prediction error sign interactions (1 < BF_incl_ < 3, see Table 5).

**Table 5:** Inclusion Bayes Factors (BF_incl_) from Bayesian repeated measures ANOVAs at ventral striatal peak voxels showing main effects of model-based prediction error (PE) across groups (see Table 4).


EFFECTS	LEFT VENTRAL STRIATUM [–10 6 –10]	RIGHT VENTRAL STRIATUM [10 12 –12]

PE sign	1060.335	7059.337

Group	0.878	0.719

Group * PE Sign	1.966	1.759


## Discussion

Here we comprehensively examined the computational underpinnings of reinforcement learning impairments in a gambling group (n = 23, n = 7 fulfilling one to three DSM 5 criteria for gambling disorder, n = 16 fulfilling four or more criteria) and a matched control group (n = 23), using a combination of computational modeling and functional magnetic resonance imaging (fMRI). Accuracy on the learning task was substantially reduced in the gambling group, whereas there was little credible evidence for group differences in response times (RTs). Computational modeling revealed that in both groups, extended reinforcement learning drift diffusion models (RLDDMs) in which both non-decision time and boundary separation (decision threshold) were modulated across trials according to a power function provided a superior account of the data (see below for discussion). The model with the best numerical fit (RLDDM8) showed good parameter and model recovery, and accurately reproduced the observed accuracy and response time (RTs) changes over the course of learning in both groups. Computational modeling revealed three major group differences: Compared to the control group, the gambling group exhibited shorter non-decision times, a more rapid reduction of decision thresholds over the course of learning, and a reduced value modulation of the drift rate. Neuroimaging analyses replicated effects of value in ventromedial prefrontal cortex, and prediction error in ventral striatum. However, Bayesian analyses revealed that evidence for group differences in these effects was at most anecdotal.

Model comparison showed that, numerically, RLDDM8 (a model with dual learning rates, and modulated non-decision time and decision threshold according to power functions) exhibited the best fit in both groups. However, there was some overlap in the 95% confidence intervals of the -elpd difference between RLDDM8 and the second best models in both groups. The runner-up model also differed between groups (RLDDM6 for the control group, RLDDM4 for the gambling group), such that, overall, the model comparison was somewhat inconclusive. We nonetheless chose to focus subsequent analyses on the RLDDM8, for the following reasons. First, the runner-up models in both groups were nested versions of RLDDM8. In such cases, an estimation approach (rather a categorical model comparison) can be more informative (Kruschke, 2015), as it allows a quantification of the degree of evidence that nested parameters (such as *α_exp_, τ_exp_*) are different from zero. Indeed, despite the inconclusive model comparison, *τ_exp_* was reliably < 0 in both groups. Second, model recovery analyses revealed that recovery was in fact best for RLDDM8. Third, parameter recovery simulations confirmed that, despite its’ greater complexity, RLDDM8 parameters could be reliably recovered (see below). The model ranking differences between groups mostly recapitulate what can be observed from the analysis of the posterior distributions. For example, the top four models in the gambling group all allowed for a modulated boundary separation parameter. This contrasts with the control group, where models ranked third and forth included a fixed boundary separation, resonating with the results from the analysis of posterior distributions, which revealed that the decay of the decision threshold was more consistent in the gambling group compared to the control group (see below). Overall, -elpd scores were substantially lower in the control group compared to the gambling group. This is likely a consequence of the fact that accuracy was overall lower in the gambling group compared to the control group, such that the RL model provided a poorer account of the data – decisions were noisier with respect to the RL model in the gambling group. This account is also consistent with the control analyses using a standard softmax model (see Supplemental Figure 10 and Supplemental Table 3), which revealed increased decision noise (a lower inverse temperature parameter) in the gambling group.

We performed extensive checks to verify the performance of RLDDM8. First, we ran a series of parameter recovery simulations, which revealed that both subject-level and group-level parameters recovered well. Parameter recovery essentially determines the upper bound of reliability. It is therefore reassuring that estimated subject-level parameters showed a correlation between .59 and .90 with the true generating parameters. Likewise, estimated posterior distributions of group-level parameters generally contained the true generating parameters within their 95% highest posterior density intervals (Fontanesi, Gluth, et al., 2019). RLDDM8 also showed satisfactory model recovery performance, which was numerically better than both RLDDM4 and RLDDM6. Second, model performance was verified in a series of posterior predictive checks. In both groups, RLDDM8 reproduced both the increases in accuracy and the decreases in RTs over trials well. The requirement of including modulated decision thresholds and non-decision times was particularly evident in the gambling group, where a simpler model without modulated decision threshold (RLDDM1) failed to fully account for the reductions in RTs over trials. RLDDM8 also reproduced both individual-participant RT distributions as well as RT changes over trials in individual participants.

Analysis of model parameters then allowed us to examine group differences in computational processes underlying task performance. Non-decision times, reflecting aspects of the RT that are unrelated to the evidence accumulation process, showed a similar decay over time in both groups, but the non-decision time offset τ_0_ was substantially lower in the gambling group. In contrast, the decision threshold showed a substantially more rapid decay in the gambling vs. the control group (α_exp_ was reliably more negative). That is, over the course of the experiment, individuals from the gambling group, more than controls, increasingly shifted their focus from accuracy to speed. These findings converge with previous observations of other forms of maladaptive decision-making and action selection in gambling disorder, such as increased motor impulsivity (Chowdhury et al., 2017), higher urgency/reduced premeditation (Kräplin et al., 2014) and higher levels of temporal discounting (MacKillop et al., 2011; Wiehler & Peters, 2015). Attenuated deliberation during decision-making is also reminiscent of previous findings of impaired goal-directed control during RL in disordered gambling (Bruder et al., 2021; Wyckmans et al., 2019). Similar effects have been shown to contribute to gambling behavior in laboratory settings (Kim et al., 2022; Shao et al., 2013). However, further work is required to more directly link such processes to maladaptive gambling behavior as it occurs in real life settings. In addition to alterations in decision thresholds, performance deficits in the gambling group were linked to a substantial reduction in the modulation of the drift rate by Q-value differences. Taken together, our findings highlight the power and utility of computational analyses via RLDDMs (Miletić et al., 2020): model-based decomposition of RT distributions revealed substantial group differences in component processes underlying reinforcement learning and action selection, despite the fact that overall RTs were similar between groups. Note that these group differences were also in part reflected in the group differences in model ranking amongst the runner-up models to RLDDM8 (see above).

A previous preprint version of the present manuscript also reported group differences for the model with a single learning rate (RLDDM4). While this analysis revealed highly similar results for τ_0_ and α_exp_, the drift rate modulation parameter v_coeff_ was not reliably different in the two groups in that model, contrasting with the results for RLDDM8. This discrepancy is likely due to interactions between learning rates and drift rate modulation. The mean difference in learning rates was more variable in the gambling group (Supplemental Figure 9), and generally, mean posterior estimates of learning rate standard deviations were higher in the gambling group compared to the control group (gambling group: *M_SD_, η*_+_ = 2.079, *M_SD_, η*_–_ = 1.395; control group: *M_SD_, η_+_* = 1.452, *M_SD_, η*_–_ = .8185, all values reported in units of learning rates in standard normal space). In some individuals from the gambling group, these effects may therefore have led to a misestimation of Q-values in RLDDM4, where learning rates are forced to be the identical regardless of feedback type. The similar v_coeff_ parameters in the two groups in RLDDM4 may then be due to the inaccurate Q-value estimates in some in participants from the gambling group. At the same time, lower learning rates were observed in the gambling group in RLDDM4, but these differences were not reliable in RLDDM8 (although, numerically mean learning rates were also lower in the gambling group in RLDDM8). This difference could result from increased estimation noise in RLDDM8, where effects are split amongst two parameters.

These results might provide some insights into potential neurocomputational mechanisms underlying the development and maintenance of gambling behavior. In animal models, exposure to uncertainty gives rise to behavioral and neural effects similar to those observed during repeated exposure to drugs of abuse (Anselme et al., 2013; M. J. F. Robinson et al., 2014, 2015; Zack et al., 2014), conceptually linking behavioral addictions (M. J. F. Robinson et al., 2016) and theories of substance-use-disorders such as incentive sensitization theory (T. E. Robinson & Berridge, 1993). In structured environments, overall experienced uncertainty is inversely related to learning performance, and midbrain dopamine neurons fire maximally during uncertain reward prediction (Fiorillo et al., 2003). Likewise, human subcortical dopaminergic structures encode risk (Preuschoff et al., 2006), and striatal dopamine release in gambling disorder is highest under conditions of maximum uncertainty (Linnet et al., 2012). There is also some evidence that gambling disorder might be linked to an overall increase in dopamine availability in the striatum (van Holst et al., 2018). Therefore, one could speculate that an increase in overall uncertainty and concomitant dopamine release (Fiorillo et al., 2003), combined with a potentially general increase in dopamine levels in the gambling group (van Holst et al., 2018), might underlie the observed effects. In line with this interpretation, decision threholds were reduced by pharmacologically increasing dopamine levels using the same task reported here (Chakroun et al., 2023). Likewise, the dopamine precursor tyrosine reduced decision thresholds across two different decision-making tasks (Mathar et al., 2022).

In previous work (Wiehler et al., 2021), we examined exploration during reinforcement learning using a restless four-armed bandit task (Daw et al., 2006) in the same group of participants. This previous task differs from the present reinforcement learning task in a number of important respects: First, average payoffs of each bandit changed continuously according to gaussian random walk processes, whereas in the present task, reinforcement rates were stable. Second, reward feedback consisted of points in the range of 0–100, whereas in the present task, participants received probabilistic binary (win / no win) feedback. Third, 300 trials in total were performed in the bandit task, whereas the present task was substantially shorter. Finally, in our previous task, a stricter response deadline was included, which precluded us from comprehensively analyzing RTs and sequential sampling models. Interestingly, however, in the four-armed bandit task, performance was similar between the gambling and the control group (Wiehler et al., 2021). Yet, computational modeling revealed that the gambling group relied less on a “directed exploration” strategy (Wiehler et al., 2021) that favours selection of uncertain options for information gain (Wilson et al., 2021). It is nonetheless striking that in the arguably more complex task in a volatile environment, impairments in the gambling group were more subtle, whereas in the present stationary task, group differences in overall accuracy were substantial. What could account for these relative differences in performance? One possibility is that the required degree of temporal integration plays a role. In the restless bandit task, reward feedback on any given trial provides (almost) complete information on the current value of a chosen bandit (“almost” because outcomes are corrupted by gaussian observation noise). In contrast, in order to accurately estimate the underlying reinforcement rates in the present task, binary outcomes need to be integrated across consecutive trials, which might contribute to the impairments in the gambling group. However, working memory deficits, which might contribute to impairments in feedback integration across trials, are not typical neuropsychological characteristics of gambling disorder (Kapsomenakis et al., 2018; Ledgerwood et al., 2012). A second possibility is that group differences might be more evident in the shorter RL task reported here, because group differences might be restricted to earlier trials. This was indeed the case for RTs, whereas accuracy was lower in the gambling group across all trials (compare Figures 3 and 4). A third possibility is that the two tasks may have been differentially affected by task order and/or fatigue effects. All participants completed the four-armed bandit task prior to the RL task reported here, and it thus cannot be ruled out that the gambling group may have been more affected by fatigue than the control group.

FMRI analyses across groups then confirmed 1) a positive correlation between activity in vmPFC and the average Q-value across options, which is in line with a wealth of previous imaging findings (Bartra et al., 2013; Chib et al., 2009; Clithero & Rangel, 2014; Plassmann et al., 2007), including results from the same task (Chakroun et al., 2023). Likewise, reward prediction error effects were replicated in bilateral ventral striatum and ventro-medial prefrontal cortex. However, for both effects, Bayesian analyses revealed at best anecdotal evidence for group differences. Alterations in regions of the reward system, in particular ventral striatum and ventro-medial prefrontal cortex, have frequently been reported in gambling disorder, as outlined in a number of reviews (Clark et al., 2019; Fauth-Bühler et al., 2017; Potenza, 2013). However, the directionality of these changes has long puzzled researchers, as both increases and reductions have been reported (Balodis et al., 2012; Clark et al., 2019; Leyton & Vezina, 2012, 2013; van Holst, Veltman, van den Brink, et al., 2012), e.g. depending on task phases (Clark et al., 2019; van Holst, Veltman, Büchel, et al., 2012), contextual factors (Leyton & Vezina, 2013; Miedl et al., 2014) or reinforcer categories (Miedl et al., 2012; Sescousse et al., 2013). Although, numerically, the contrast between positive and negative prediction errors in bilateral striatum appeared to be somewhat more pronounced in the gambling group, which would be consistent with some earlier observations (Clark et al., 2019), evidence was only anecdotal (1 < BF_incl_ < 3). While the replication of core previous results in vmPFC and VS increases the confidence in the fMRI results, several reasons may underlie the lack of reliable group differences. First, we focused our analysis on two core regions previously implicated in RL and disordered gambling (Clark et al., 2019), and it is therefore possible that group differences in other circuits were overlooked. Second, one study with a substantially larger sample size only observed effects in gambling disorder when depressive symptoms were also taken into account (Fauth-Bühler et al., 2014), suggesting that fMRI effects might in some cases be restricted to specific gambling disorder subgroups. Finally, more general reliability issues with fMRI contrasts (Fröhner et al., 2019) might contribute to the overall heterogeneity in the field.

A number of limitations of the present study need to be acknowledged. First, the sample size was relatively small, and findings thus require replication in larger samples. However, modeling used hierarchical Bayesian estimation procedures that are suitable for cases of limited observations, and parameter and model recovery checks confirmed this. Second, as is often the case in studies on disordered gambling, due to the higher prevalence of problem gambling in males (Hing et al., 2016), the gambling group only includeed male participants, limiting the generalizability of our results. Third, in contrast to the original study according to which the task was set up (Pessiglione et al., 2006), we only included a gain condition, and no loss condition. The degree to which the reported impairments in reinforcement learning in the gambling group extend to tasks with an explicit loss condition therefore remain to be examined in future studies. Fourth, a classification of individuals suffering from disordered gambling into different subtypes according to clinical characteristics, disorder trajectories and/or gambling motivations have been proposed (Blaszczynski & Nower, 2002; Milosevic & Ledgerwood, 2010). These factors potentially reflect important individual differences in the context of disordered gambling, and the same holds for the preferred gambling format of individuals. However, given the small sample size, examination of such subtypes as well as effects of preferred gambling format was not feasible. Finally, comborbidities are potentially important confounds in studies on disordered gambling. Although groups were matched on alcohol use and smoking in the present study, depression symptoms were higher in the gambling group, which is a common finding (Dowling et al., 2017). Depression is known to be associated with RL impairments (Mukherjee et al., 2023; Pike & Robinson, 2022), and depression symptoms might thus confound some of the observed group differences. However, the most consistent finding in depression are alterations in learning rates (Pike & Robinson, 2022), contrasting with the primary group differences in terms of decision threshold modulation and non-decision times that we report here. Nonetheless, future studies might benefit from a more comprehensive assessment of comorbidities than done here.

Taken together, we provide a comprehensive model-based analysis of computational mechanisms underlying impaired reinforcement learning performance in gambling disorder. Model-based decomposition of RTs revealed that, although overall RTs were similar between groups, the underlying processes differed considerably. In particular, the gambling group showed shorter non-decision times, an increasing focus on speed vs. accuracy over the course of the experiment (reduction of decision thresholds over time) and a reduced impact of Q-value differences on the drift rate. These findings highlight that reinforcement learning impairments in gambling disorder are likely attributable to alterations in multiple component processes.

## Data Accessibility Statement

Raw fMRI data cannot be shared publicly because participants did not provide consent for having raw imaging data posted in a public repository. JAGS model code, behavioral data and imaging data from peak voxels are available on the Open Science Framework (https://osf.io/mb8zr/). Processed fMRI data (T-maps) for the effects shown in Figures 6 and 7 are also available on OSF (https://osf.io/mb8zr/).

## Additional File

The additional file for this article can be found as follows:

10.5334/cpsy.104.s1 Supplemental material. Supplemental figures and tables.
