# Supplementary material for: Decomposition of Reinforcement Learning Deficits in Disordered Gambling via Drift Diffusion Modeling and Functional Magnetic Resonance Imaging
Source: Comput Psychiatr. 2024 Mar 20;8(1):23–45. doi: 10.5334/cpsy.104 (PMC11104325; doi:10.5334/cpsy.104)
Supplement: Supplemental material. — Supplemental figures and tables. [file cpsy-8-1-104-s1.pdf]

Wiehler & Peters: Decomposition of reinforcement learning deficits in disordered gambling via drift diffusion modeling and functional magnetic resonance imaging.

Supplemental material

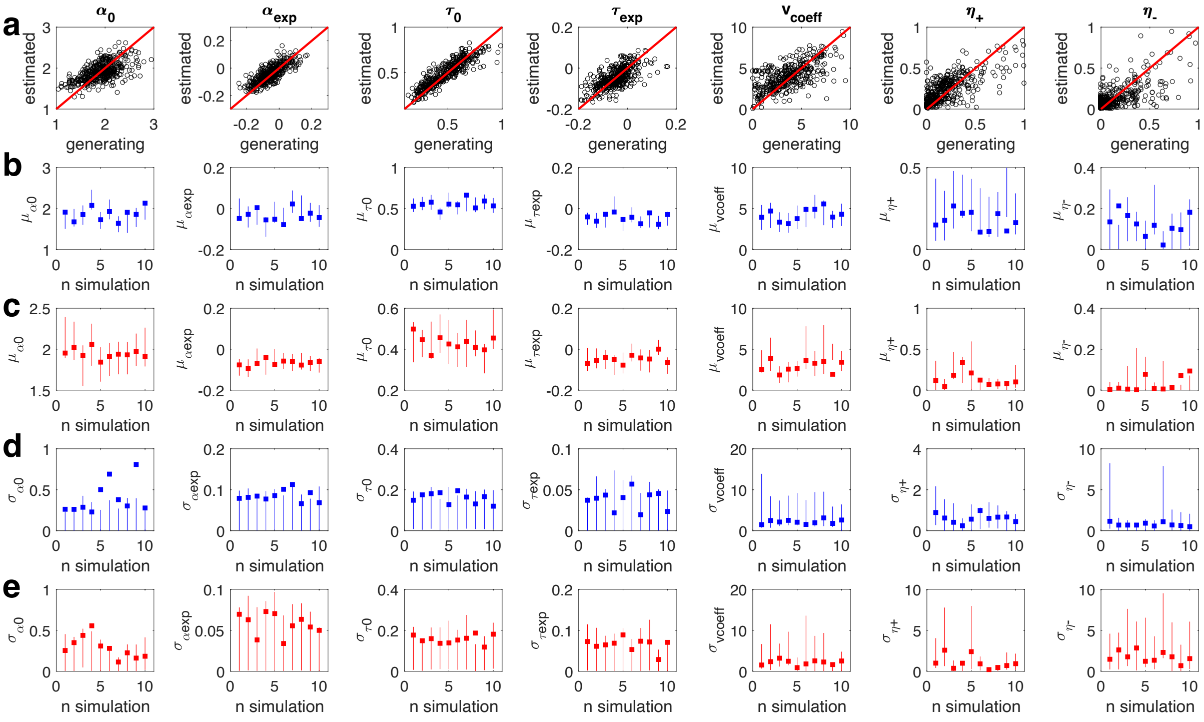

Supplemental Figure 1. Parameter recovery simulation results for RLDDM8 (dual learning rates, modulated decision threshold and non-decision time). a) Generating vs. estimated single-subject parameters across all 10 simulations. b) Control group parameter means. c) Gambling group parameter means. d) Control group parameter standard deviations. e) Gambling group parameter standard deviations. In b-e, squares denote the generating parameter, and vertical lines denote the 95% highest posterior density of the parameter estimation.

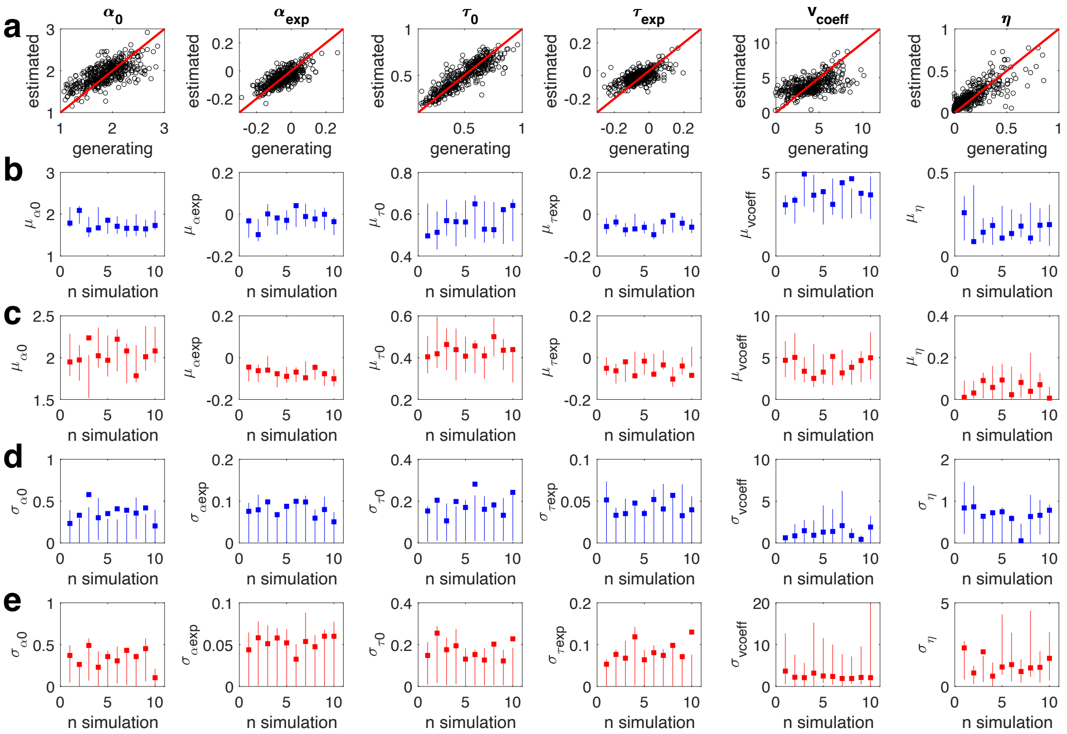

Supplemental Figure 2. Parameter recovery simulation results for RLDDM4 (single learning rate, modulated decision threshold and modulated non-decision time). a) Generating vs. estimated single-subject parameters across all 10 simulations. Panels b-e show generating group-level parameters (means and standard deviations) plotted as squares, and estimated 95% highest posterior density intervals as vertical lines. b) Control group parameter means. c) Gambling group parameter means. d) Control group parameter standard deviations. e) Gambling group parameter standard deviations.

Supplemental Table 1. Pearson correlations between generating and estimated individual-participant parameters (pooled across 10 simulations) for the RLDDM4 and RLDDM8.

Parameter	RLDDM4	RLDDM8
$\boldsymbol{\alpha}_0$	.70	.59
$\boldsymbol{\alpha}_{exp}$	.73	.78
$\boldsymbol{\tau}_0$	.88	.90
$\boldsymbol{\tau}_{exp}$	.65	.64
$\boldsymbol{v}_{coeff}$	.57	.69
$\boldsymbol{\eta}$	.83	-
$\boldsymbol{\eta}_+$	-	.71
$\boldsymbol{\eta}_-$	-	.69

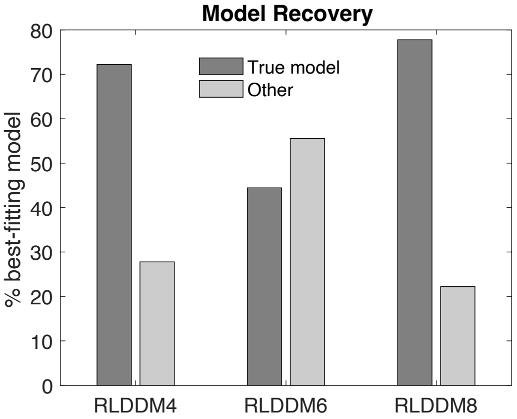

Supplemental Figure 3. Model recovery results. In addition to the best-fitting model (RLDDM8), model recovery focused on those models that exhibited overlap with RLDDM8 in terms of the 95%CI of the -elpd score in at least one group. This was the case for RLDDMs 4 and 6. We simulated n=20 full datasets from each of the three models, and re-fit the simulated data with all nine models from our model space. Plotted is the percentage of simulations in which the true data-generating model was recovered (*True model*) and the percentage of simulations in which some other model accounted for the data best (*Other*). Recovery was successful in > 70% of simulatons for both RLDDM4 and RLDDM8, whereas it was <50% for RLDDM6. Note that chance level is 11.11%.

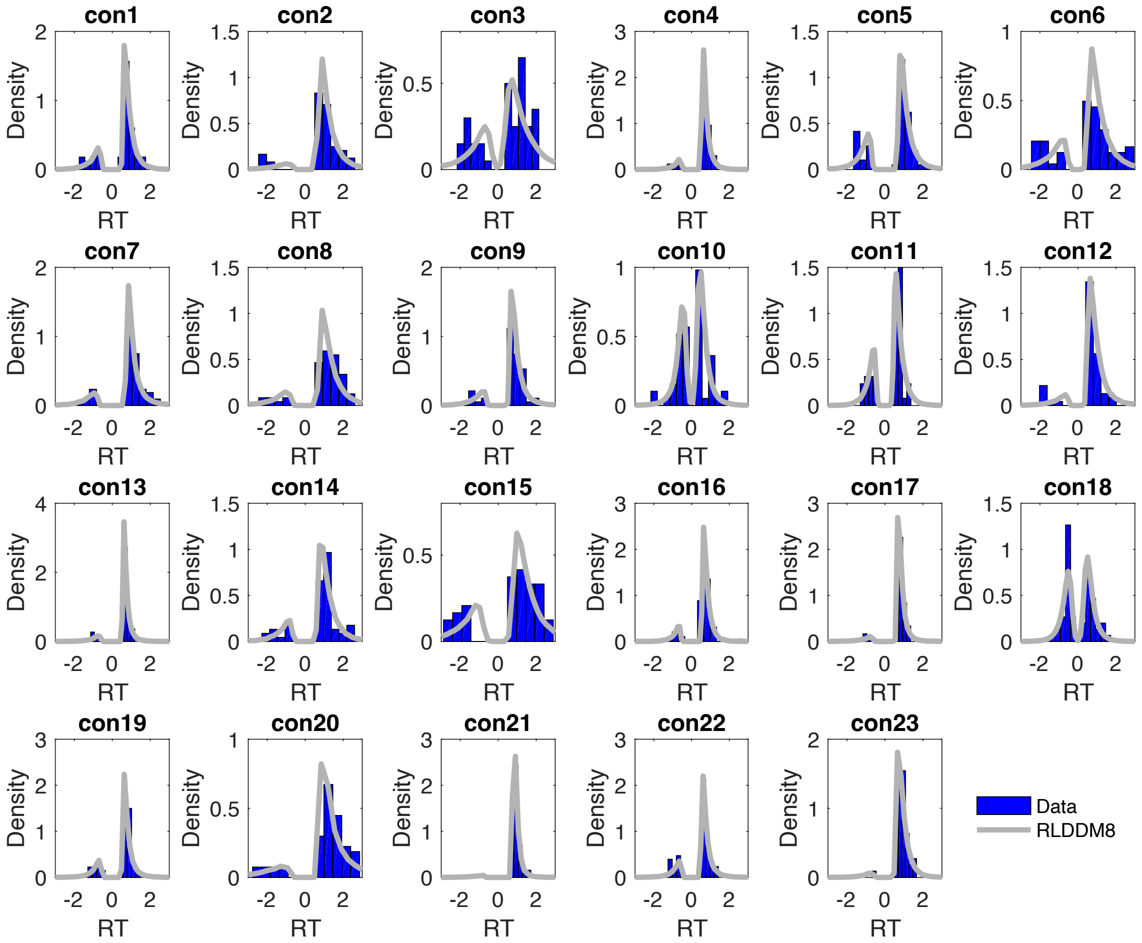

Supplemental Figure 4. Individual subject posterior predictive checks for the control group and RLDDM8. Plotted are individual-subject observed RT distributions (blue histograms) and model simulated RT distributions (grey lines, smoothed histograms of 1k RT distributions simulated from the model's posterior distribution.

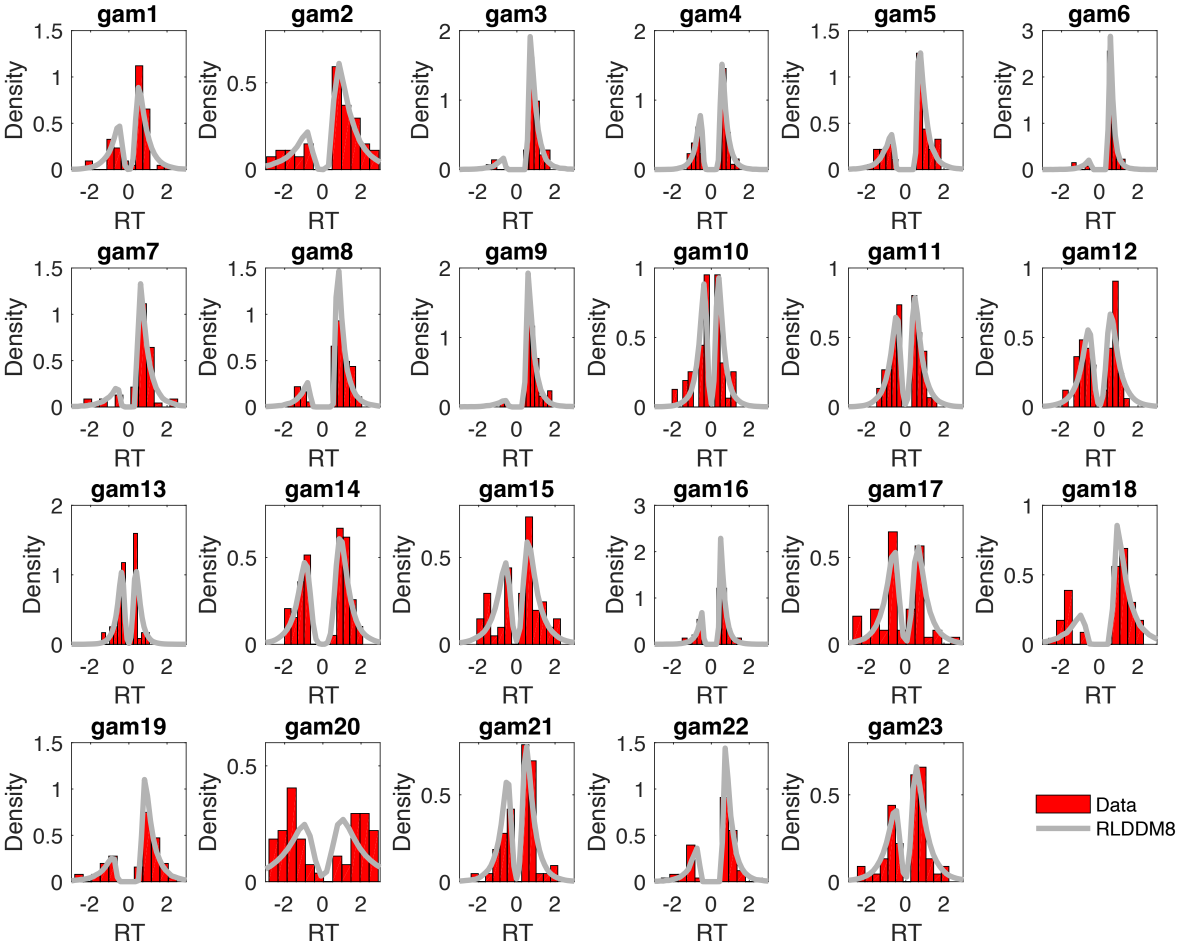

Supplemental Figure 5. Individual subject posterior predictive checks for the gambling disorder group and RLDDM8. Plotted are individual-subject observed RT distributions (red histograms) and model simulated RT distributions (grey lines, smoothed histograms of 1k RT distributions simulated from the model's posterior distribution.

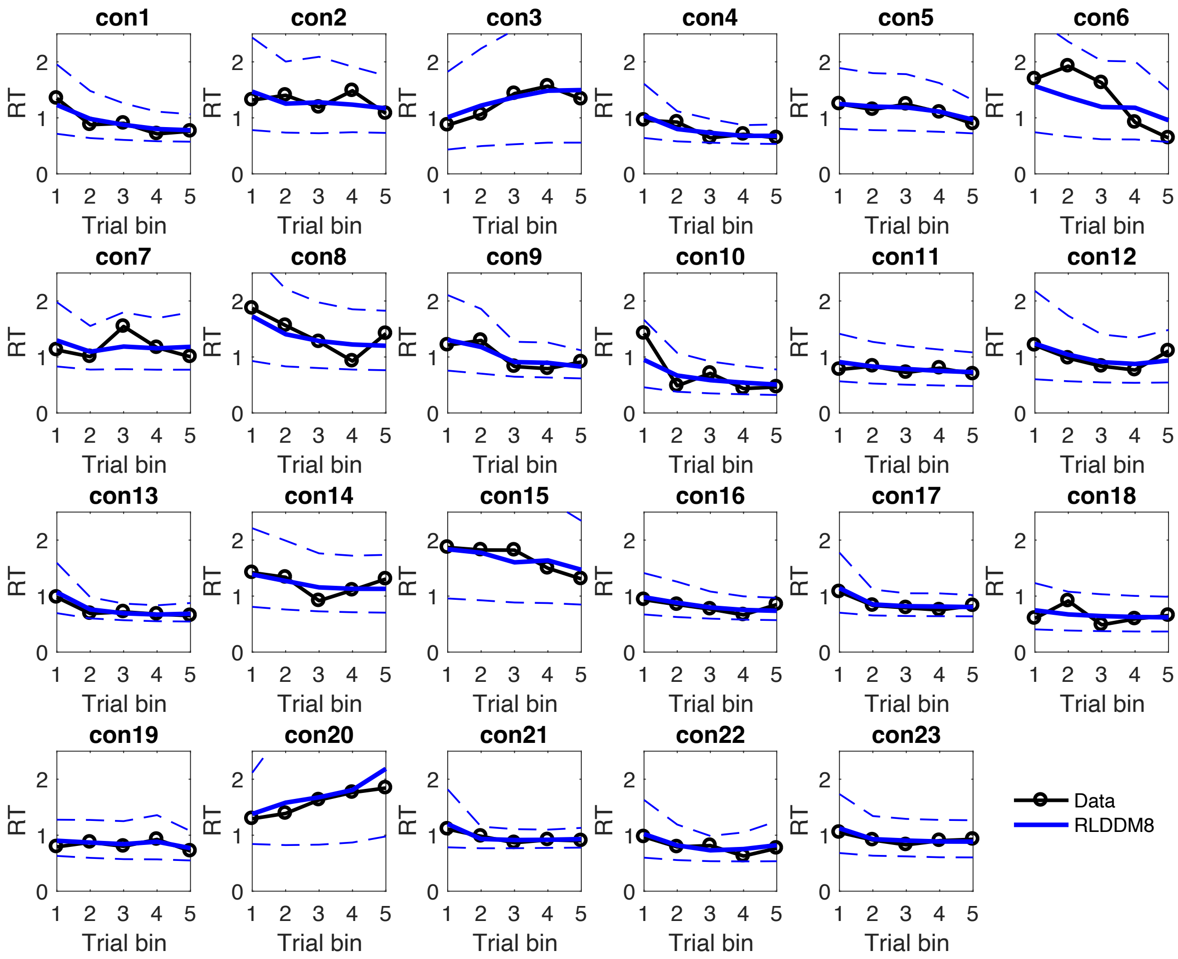

Supplemental Figure 6. Posterior predictive checks for RT changes over the course of learning in individual control group participants. Black lines denote observed mean RTs per trial bin. Solid blue lines denote mean RTs across 1k simluated data sets from the RLDDM8 posterior distribution. Dashed lines denote the +/- 95% percentile of the simulated RTs.

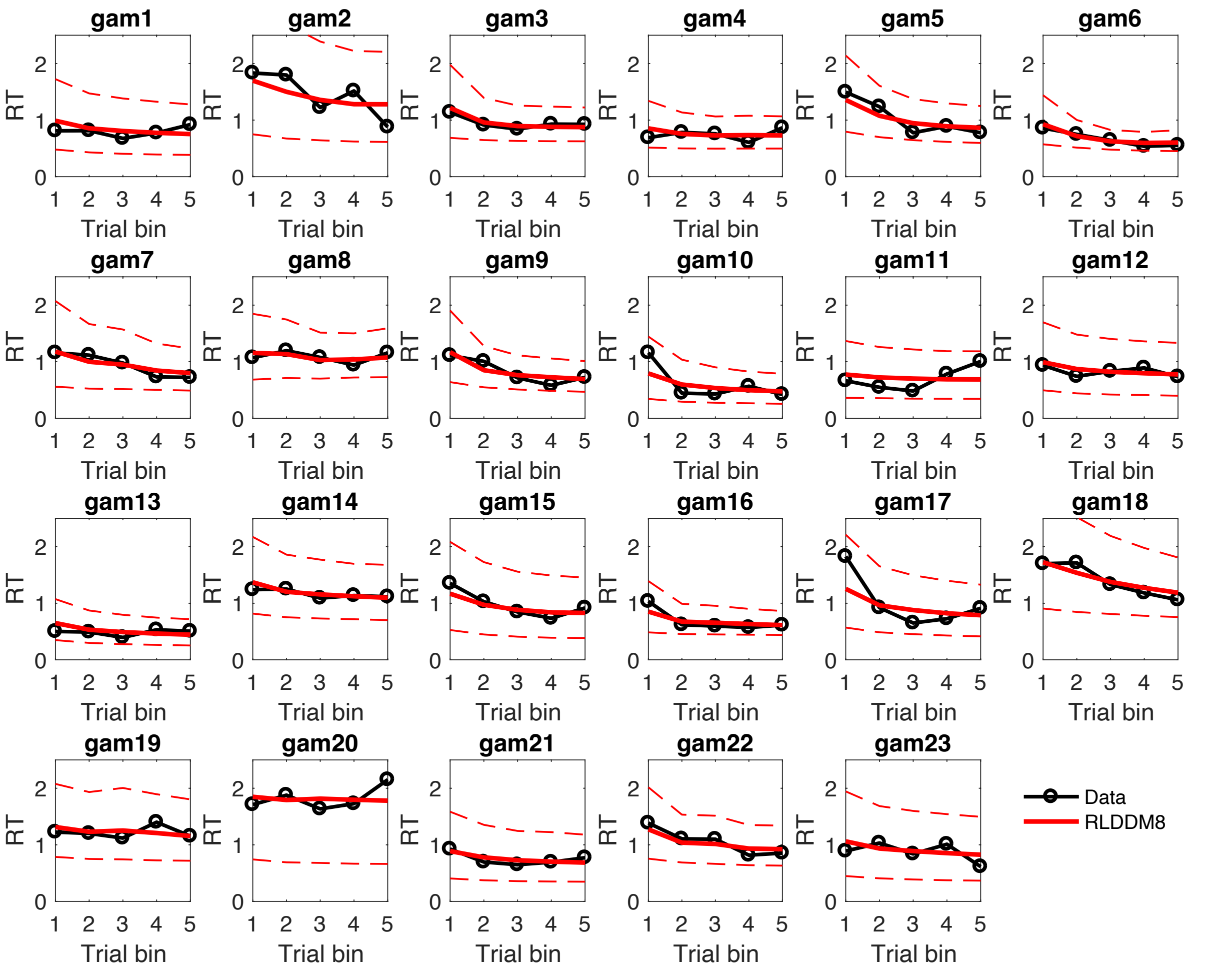

Supplemental Figure 7. Posterior predictive checks for RT changes over the course of learning in individual gambling disorder group participants. Black lines denote observed mean RTs per trial bin. Solid red lines denote mean RTs across 1k simluated data sets from the RLDDM8 posterior distribution. Dashed lines denote the +/- 95% percentile of the simulated RTs.

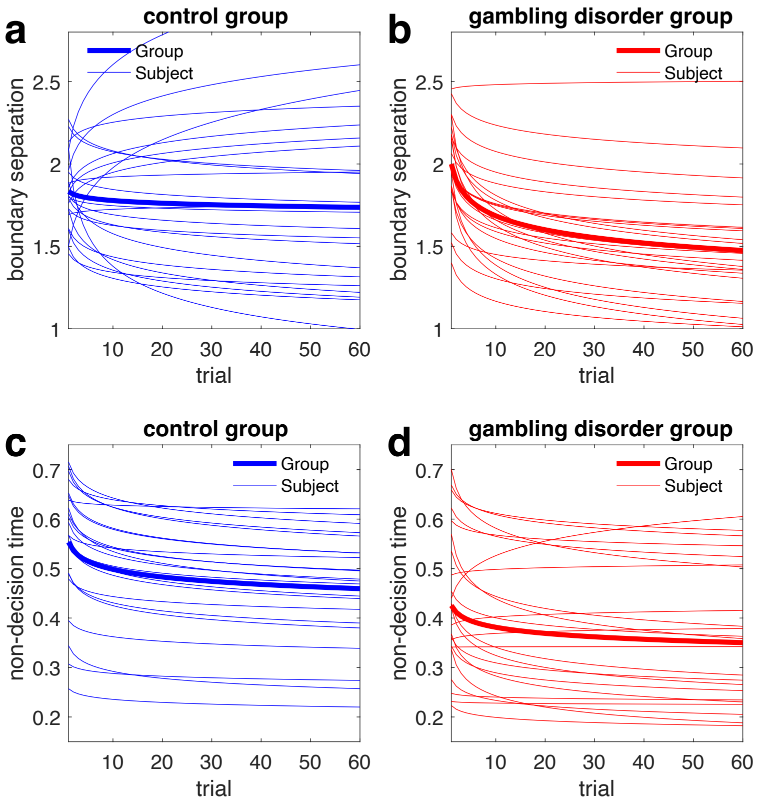

Supplemental Figure 8. Estimated trial-wise decision threshold (a,b) and non-decision time parameters (c,d) for control group participants (blue) and participants from the gambling group (red) according to RLDDM8. The solid lines plot parameter changes based on the mean group-level posteriors, whereas the thin lines depict individual subject curves.

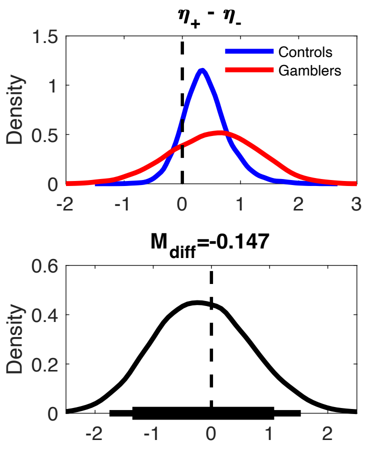

Supplemental Figure 9. Upper panel: Posterior differences between positive and negative learning rates per group (controls: blue, gamblers. red). Lower panel: posterior group difference in learning rate differences (controls – gamblers). Solid (thin) horizontal lines in the lower panel denotes 85% (95%) highest posterior density interval.

Supplemental Table 2. Learning rate differences in the dual learning rate model RLDDM8 (mean posterior group difference (M_{diff}) and Bayes factors testing for directional effects, dBF). dBF values > 1

quantify the degree of evidence for a reduction in a parameter in gamblers vs. controls compared to the evidence for an increase. dBF values < 1 reflect the reverse. Directional test refers to tests for directional effects of the learning rate difference performed separately per group.

**RLDDM 8 parameter**	**Group difference**		**Directional test (dBF)**	
	M_{diff}	***dBF***	***Controls***	***Gamblers***
$\eta_{+} - \eta_{-}$	-.147	.79	6.32	3.37

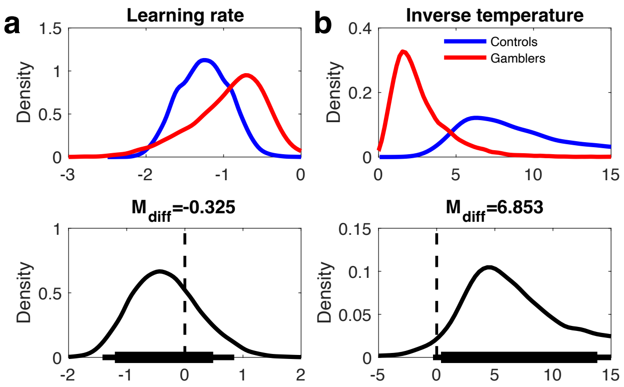

Supplemental Figure 10. Upper panels: Softmax model posterior distributions of group mean learning rates (a) and softmax inverse temperatures (b) for controls (blue) and gamblers (red). Lower panels: posterior group differences per parameter (controls – gamblers). Solid (thin) horizontal lines in the lower panels denote 85% (95%) highest posterior density intervals. Note that learning rates were fitted in standard normal space [-3, 3] as plotted here, and were back-transformed to the interval [0, 1] via the inverse cumulative normal distribution function.

Supplemental Table 3. Group differences: Mean posterior group differences in model parameters (M_{diff}) and Bayes factors testing for directional effects (dBF). dBF values > 1 quantify the degree of evidence for a reduction in a parameter in gamblers vs. controls compared to the evidence for an increase. dBF values < 1 reflect the reverse.

**SM parameter**	**Group difference**	
	M_{diff}	***dBF***
η	-.325	.373
β	6.85	22.89
